# Nash Bargaining-Based Hybrid MAC Protocol for Wireless Body Area Networks

**DOI:** 10.3390/s26030967

**Published:** 2026-02-02

**Authors:** Haoru Su, Jiale Yang, Rong Li, Jian He

**Affiliations:** College of Computer Science, Beijing University of Technology, Beijing 100124, China; suhaoru@bjut.edu.cn (H.S.); white@emails.bjut.edu.cn (J.Y.); leerong@bjut.edu.cn (R.L.)

**Keywords:** wireless body area network, medium access control, cooperative game, eHealth, internet of things

## Abstract

Wireless Body Area Network (WBAN) is an emerging medical health monitoring technology. However, WBANs encounter critical challenges in balancing reliability, energy efficiency, and Quality of Service (QoS) requirements for life-critical medical data. The design of its Medium Access Control (MAC) protocol has challenges since dynamic body-shadowing effects and heterogeneous traffic patterns. In this paper, we propose the Nash Bargaining Rate-optimization MAC (NBR-MAC), a hybrid MAC protocol that integrates TDMA-based Guaranteed Time Slots (GTS) with CSMA/CA-based contention access. Unlike traditional schemes, we model the rate allocation as an Asymmetric Nash Bargaining Game, introducing a rigorous disagreement point to guarantee minimum service for critical nodes. The utility function is normalized to resolve dimensional inconsistencies, incorporating sensor priority, buffer status, and channel quality. The Nash Bargaining solution is derived after proving convexity and verifying the axioms. Superframe time slots are allocated based on sensor data priority. Simulation results demonstrate that the proposed protocol enhances transmission success ratio and throughput while reducing packet age and energy consumption under different load conditions.

## 1. Introduction

Global population aging and escalating chronic disease prevalence are placing unprecedented strain on traditional healthcare systems, as conventional monitoring approaches fall short of the urgent demand for real-time, continuous health surveillance. This gap underscores the critical need for innovative technologies that enable early disease warning and proactive health management. Wireless Body Area Networks (WBANs), integrating wearable and implantable sensors with smart terminals via short-range wireless communication, emerge as a pivotal enabler for intelligent, personalized healthcare monitoring beyond spatial and temporal constraints [1]. Besides healthcare, WBAN can also be applied in the fields of military, industry, emergency services, rehabilitation, special assistance, sports, entertainment, fitness, consumer electronics, and so on.

**Figure 1:**
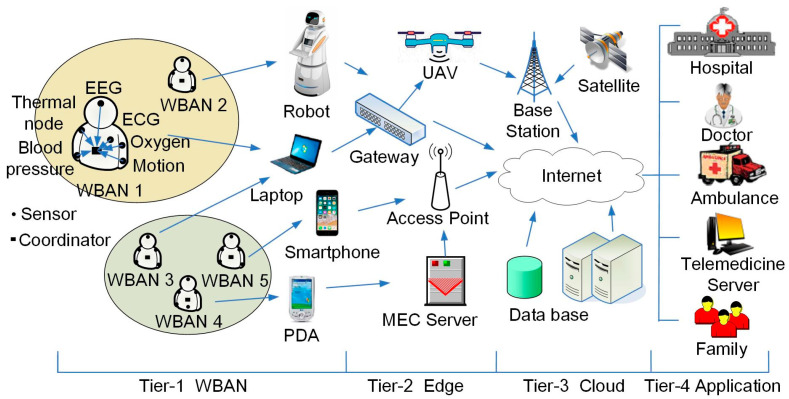
System architecture of general eHealth system based on WBANs.

The general network architecture of an eHealth system based on WBANs is typically divided into four tiers, each responsible for different functions to ensure the efficiency and reliability of data transmission and management [2], as shown in Figure 1.

The first tier is WBAN, which comprises wearable or implantable sensors that continuously acquire physiological signals, such as heartbeats, body temperatures, EMG, EEG, and ECG. Motion sensors extract discriminative features to classify various postures and movements, including lying, sitting, walking, and running. Sensed data are transmitted to the WBAN coordinator or other personal center devices, such as a smartphone, PDA, laptops, or service robot. Commonly adopted short-distance wireless communication technologies encompass Wi-Fi, Bluetooth, and ZigBee.

The second tier is edge computing. WBANs send data through the Access Point (AP) or the gateway. MEC servers are deployed at the network edge in close proximity to end users. Unmanned Aerial Vehicles (UAVs) provide mobile and flexible computational services. This tier performs real-time preprocessing, feature extraction, and anomaly detection to ensure low-latency feedback while reducing transmission overhead.

The third tier is cloud computing. The remote cloud servers conduct scalable storage and computationally intensive analytics, including machine learning model training, statistical processing, longitudinal trend analysis, and multi-patient data fusion, provisioning backend services to upper layers.

The fourth tier is application. It delivers role-based end-user services through intuitive dashboards for clinicians and patients. It enables interactive data visualization, intelligent alert management, and AI-driven personalized health recommendations. Beyond routine monitoring, this layer facilitates remote expert consultation and collaborative decision-making via secure Internet channels. Critically, it integrates automated emergency response protocols: upon detecting life-threatening events (e.g., myocardial infarction), the system instantaneously triggers location-aware alerts to the nearest emergency medical services, ensuring rapid clinical intervention.

A number of communication standards are suitable for WBANs, including the IEEE 15.4 [3] and the IEEE 802.15.6 [4]. The IEEE 802.15.4 standard is a widely recognized specification for Low-Rate Wireless Personal Area Networks (LR-WPANs), designed to support low-complexity, low-power consumption, and low-cost devices. The IEEE 802.15.6 standard is designed for WBANs, providing low-power communication solutions for both medical and non-medical applications. The standard defines three physical layers (PHY): narrowband (NB), ultra-wideband (UWB), and human-body communication (HBC), each tailored to different application requirements.

The Medium Access Control (MAC) protocol plays a crucial role in ensuring efficient and reliable communication within WBANs. The primary function of the MAC protocol is to coordinate collision-free data transmission among multiple nodes within the same network. In WBANs, different sensor nodes undertake various sensing tasks, and thus, the transmission rate and delay requirements for each sensor to transmit collected data to the coordinator vary. The MAC protocol needs to assign appropriate access schemes to the nodes in the network and schedule the optimal transmission timing based on the QoS requirements of different sensors, thereby avoiding collisions when different nodes transmit control frames and data frames.

To address these issues, this paper proposes a Nash Bargaining Rate-optimization MAC (NBR-MAC) protocol for Wireless Body Area Networks, a hybrid MAC protocol that integrates TDMA-based Guaranteed Time Slots (GTS) with CSMA/CA-based contention access. We formulate the transmission rate optimization problem as a cooperative game. The utility function is normalized to resolve dimensional inconsistencies, incorporating sensor priority, buffer status, and channel quality. Then, we prove the convexity and verify the four axioms of the Nash Bargaining Solution (NBS). The NBS is derived by convex optimization problem. After that, the superframe structure is introduced. The time-slot sharing strategy is designed based on the data priority of the sensor nodes. Simulation results exhibit that the proposed protocol improves the network performance, including transmission success ratio, throughput, packet age, and energy consumption.

The remainder of this paper is organized as follows. In Section 2, we review the related studies. Section 3 presents the NBR-MAC protocol. In Section 4, we discuss the performance evaluation. Conclusions and future work are presented in Section 5.

## 2. Related Work

Since MAC protocol is the essential for WBANs, numerous MAC protocols have been proposed [5].

Dam et al. [6] propose the T-MAC, a contention-based MAC protocol, with an adaptive duty cycle by dynamically ending the active part of it to reduce energy consumption. Touijer et al. [7] propose a backoff counter selection mechanism and a contention window adjustment strategy, validating their effectiveness using the UPPAAL-SMC model checking tool. Experimental results showed that this method reduced collisions, improved data transmission success ratio and energy efficiency, and optimized the communication performance of WBANs. Liang et al. [8] propose a Priority Ladder Resource Scheduling (PLRS) scheme, which optimizes resource scheduling. This is achieved through multi-level priority division, ladder slot allocation, and interval-based task ordering. It divides data priorities into four levels and adopts a round-by-round slot allocation method to ensure timely data transmission for high-priority nodes while improving network fairness. Shunmugapriya et al. [9] propose a three-layer hybrid architecture combining TDMA and CDMA to reduce signal interference. It uses TDMA for slot allocation to reduce interference between in-body sensors and combines CDMA coding transmission to avoid signal conflicts between different WBAN devices.

Olatinwo et al. [10] propose the SDC-HYMAC protocol, which introduces a priority-based slot allocation strategy to reduce energy consumption. It dynamically selects superframe order based on traffic information and biomedical device priority. Sakib et al. [11] introduce the EQPD protocol, designed to ensure end-to-end quality of service (QoS) by integrating the application, network, and MAC layers. It is particularly effective in heterogeneous data IoTs. Mkongwa et al. [12] propose an enhanced CSMA/CA protocol, which reconstructs the traditional fixed backoff window into four progressive intervals. It also adopts a single-phase CCA rule to improve transmission efficiency of real-time sensitive data. Saneetha et al. [13] propose a MVDR approach, which features an adaptive resource allocation system. This system includes a real-time priority module to track data stream characteristics and a predictive module that employs the Resnick formula to analyze historical data. The coordinator dynamically expands CFP length based on real-time load.

Azdad et al. [14] proposed FOTA-MAC with two versions: V1 uses a historical load-based dynamic CAP adjustment algorithm, which is suitable for scenarios with large load fluctuations such as sudden medical data. V2 adopts a traffic-isolated CAP mechanism, dividing the superframe into CAP1 for light traffic and CAP2 for heavy traffic. The coordinator adjusts the CAP partition ratio via node buffer status parsed from data frame reserved fields. Olatrinwo et al. [15] propose MDP-HYMAC based on Markov Decision Process. It models the system with a 5-tuple, solves the optimal strategy via value iteration, and uses a five-state machine model for sensors.

Ferreira et al. [16] propose CAG, which converts gait characteristics into MAC scheduling parameters by analyzing IEEE 802.15.6 beacon RSSI, and switches to default TDA mode for non-periodic movement to complement PBDT’s adaptation. Zia et al. [17] propose G-MAC, classifying buffered traffic into three priorities. Its core innovation is a dynamic resource allocation algorithm based on BSF frames (Buffer Status Frames). This algorithm operates independently of the superframe cycle, ensuring real-time resource allocation and avoiding protocol overhead interference. Al Masud et al. [18] propose Delay-tolerant MAC, reconstructing medical WBAN QoS via six-dimensional severity levels. Das et al. [19] propose CRITIC scheme, which divides the beacon cycle into dynamic EAP, fixed RAP, and dynamic MAP. Its priority scoring integrates five parameters: user priority, buffer occupancy status, packet size, data transmission rate, and packet generation rate.

Siddik et al. [20] propose E-MAC, which compresses the eight original UP levels into four and simplifies the superframe to RAP/MAP/CAP. Su et al. [21] propose MDP-MAC, which employs a reinforcement learning framework that integrates Q-learning with FDMA. The process is divided into two phases: a learning phase and an application phase. Hu et al. [22] propose PASA, which divides the sensor cycle into six coordinated phases: reservation period, activation period, energy harvesting period, upload period, and saving period. This joint optimization enables continuous operation under 0.5 W/m^2^ RF field strength. Gao et al. [23] propose BT-MAC with three superframe models. Type A for regular data using simplified B-MAP1, with nodes sleeping in inactive periods for 0.5 mW low power. Type B separating alarm frames and emergency data in EP phase (EAP + RAP). Type C adding MAP2 for unresolved collisions and using TDMA for zero-collision emergency transmission.

Wan Hassan et al. [24] proposed HB-MAC, which divides the 32-slot superframe into RAP for bursty data and MAP for deterministic ECG transmission. Rana et al. [25] propose MMH-MAC, an intelligent adaptive MAC protocol using deep learning. It employs Q-learning to adjust superframe structures, thus optimizing time-slot resources. It improves real-time performance and reliability in high-load scenarios, suitable for low-latency medical IoT. Wan Hassan et al. [26] propose Poll-based MAC. This protocol divides the superframe into three phases: Beacon, RAP, and scheduling cycle. The key innovation is dynamic polling. When nodes detect emergency data, they set the MoreData flag. The coordinator then dynamically allocates additional resources in subsequent cycles through Poll-embedded ACK frames. The i-MAC [27] devises a superframe structure that distinctly separates access for emergency and regular events. The access is scheduled based on node priority.

In [28], the authors propose EE-DCAA, a channel allocation approach which is compatible with the IEEE 802.15.6 standard. It employs both polling and contention access methods. In [29], the authors propose MG-HYMAC, a hybrid MAC protocol, in which the duty cycle operations and transmission scheduling scheme are joined to reduce the power consumption. In [30], the authors propose DeepBAN, which uses temporal convolution network (TCN)-based deep learning to predict the channel condition. The algorithm integrally considers power control, time-slot allocation, and relay node selection.

In [31], a bargaining-based optimal slot sharing (BOSS) mechanism was put forward by the authors to tackle the optimization issue related to time-slot allocation for sensor nodes. Similarly, the LTA framework [32] utilizes a multi-agent reinforcement learning (MARL) approach where each node acts as an agent, finding an optimal slot in a TDMA schedule. The decision-making is guided by a composite reward function that balances transmission delay and node temperature, making it particularly suitable for implantable devices.

To clarify the contributions of NBR-MAC, Table 1 compares it with relevant protocols.

Unlike BOSS [31], which relies on standard bargaining for slot sharing, NBR-MAC incorporates a contention period for overflow traffic and uses an asymmetric bargaining solution to explicitly favor high-urgency medical data during emergency bursts.

Existing MAC protocols for WBANs have several limitations. These protocols often struggle with energy efficiency due to issues such as idle listening, collisions, and the need for frequent synchronization. They may also fail to effectively handle the dynamic and heterogeneous traffic patterns inherent to WBANs, leading to poor spectral efficiency and increased latency. Additionally, many existing protocols lack mechanisms to prioritize emergency traffic, which is crucial for medical applications.

## 3. Nash Bargaining Rate-Optimization MAC Protocol for WBANs

We propose a Nash Bargaining Rate-optimization MAC (NBR-MAC) protocol for Wireless Body Area Networks. In this scheme, we model the transmission rate optimization problem as a cooperative game. The utility function is defined based on four sensor state factors, including transmission success ratio, buffer occupancy, buffer age, and data urgency. After proof of convexity and verification of Nash Bargaining axioms, the Nash Bargaining Solution (NBS) is calculated. The superframe is divided into four periods. The time slots within the superframe are shared based on the priority of the sensor data. The Guaranteed Time Slots (GTS) in the Contention-Free Period (CFP) are allocated to the sensor nodes with higher priority. The remining nodes transmit data in the Contention Access Period (CAP) under the CSMA/CA mechanism.

### 3.1. Network Model

In this subsection, we present the modeling of a single-hop star topology WBAN, which is a prevalent network configuration due to its simplicity and efficiency for short-range communication. In a single-hop star topology WBAN, the network architecture consists of a central coordinator and multiple sensor nodes that communicate directly with this coordinator. The sensor nodes are strategically placed to monitor various physiological parameters. The coordinator serves as the central hub, responsible for collecting data from all sensor nodes and forwarding it to a remote server or a healthcare provider. This direct communication minimizes latency and reduces the complexity of multi-hop routing, which is beneficial for real-time health monitoring applications.

**Figure 2:**
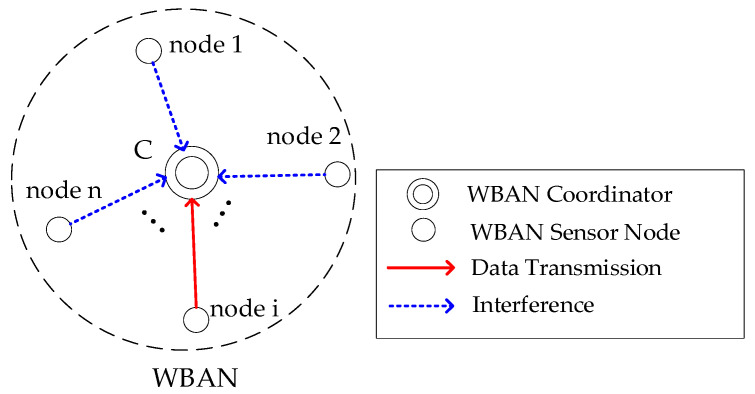
WBAN architecture with star topology.

Let ***SN*** be the set of sensor nodes in a single-hop star topology WBAN, and ***C*** be the coordinator. The network can be represented as {***SN***, ***C***}, where each node *i* ∈ ***SN*** communicates directly with ***C***. Figure 2 depicts the WBAN architecture with star topology. When node *i* transmits data to the coordinator, interference from other sensor nodes may cause collisions.

### 3.2. Cooperative Game Model

Game theory is a branch of applied mathematics that studies strategic interactions among rational decision-makers. It examines how participants make choices to maximize their own payoffs, given the strategies chosen by others. Based on whether the participants can reach a binding agreement, games can be divided into two major categories: cooperative games and non-cooperative games. If participants, starting from their own interests, reach agreements or form coalitions with other participants, it is considered a cooperative game.

Wireless Body Area Networks (WBANs) exhibit application heterogeneity, resulting in markedly diverse traffic patterns and data rate requirements. Moreover, WBAN traffic demonstrates inherent burstiness, characterized by transient intervals of significantly elevated transmission rates. Additionally, there are other QoS requirements such as latency and reliability.

In this work, we apply the cooperative game model. Potential game modeling of WBAN system is carried out from three elements of the game, which are players, strategy space, and utility function. The data transmission rate optimization game can be defined as ***G*** = <***P***, ***S***, ***U***>. ***P***, ***S***, ***U*** are players, strategy space, and utility function, respectively. Specific descriptions are as follows.

#### 3.2.1. Player

Each sensor node is regarded as a player in the game. ***P*** = ***SN*** = {1, 2, …, *N*} represents the set of sensor nodes of the WBAN. We assume sensor nodes can cooperate with each other.

#### 3.2.2. Strategy Space

The strategy space (***S***) is defined as the set of transmission rates (***R***) of sensor nodes. ***S*** = ***R*** = {*R*_1_, *R*_2_, …, *R_i_*, …, *R_N_*}. *R_i,t_* is the transmission rate of sensor node *i* at time *t*.

#### 3.2.3. Priority-Based Utility Formulation

To capture the heterogeneity of WBAN traffic, we formulate the utility function based on the Asymmetric Nash Bargaining Framework. Unlike traditional approaches that treat all nodes equally, we introduce a Dynamic Priority Weight ($\alpha_{i,t}$) for each sensor node, which serves as the exponent in the Nash Product. This weight dictates the “bargaining power” of a node and is synthesized from four key sensor states:

1. Reliability Factor ($\psi_{rel}$):

To ensure fairness, nodes with historically poor channel conditions (low transmission success ratio, $\rho_{i,t}$) should be compensated, but not to the extent of wasting bandwidth. We define the normalized reliability factor as

(1) $$\psi_{rel}=exp\left(-\frac{\rho_{i,t}}{\rho_{target}}\right)$$

where $\rho_{target}$ is the expected reliability threshold.

2. Buffer Occupancy Factor ($\psi_{buf}$)

A higher buffer occupancy indicates a risk of packet drop. Let $B_{i,t}$ be the current buffer length and $B_{i}^{max}$ be the buffer capacity. The buffer occupancy factor is modeled as

(2) $$\psi_{buf}=\frac{B_{i,t}}{B_{i}^{max}}$$

3. Data Freshness Factor ($\psi_{age}$)

For medical monitoring, data staleness can be critical. We define the age factor based on the average waiting time $A_{i,t}$ of the head-of-line packet:

(3) $$\psi_{age}=\frac{A_{i,t}}{A_{max}}$$

where $A_{max}$ is the maximum tolerance delay.

4. Data Urgency ($\psi_{urg}$)

$D_{i,t}\in 1,\ldots ,K$ represents the discrete urgency level of the physiological data (e.g., normal vs. cardiac arrest).

(4) $$\psi_{urg}=\frac{D_{i,t}}{K}$$

5. Synthesis of Priority Weight

The composite priority weight $\alpha_{i,t}$ is a weighted sum of these normalized factors:

(5) $$\alpha_{i,t}=w_{1}\psi_{rel}+w_{2}\psi_{buf}+w_{3}\psi_{age}+w_{4}\psi_{urg}$$

where $\sum w_{k}=1$ are tuning coefficients determined by the specific medical application scenario.

6. Utility Function Definition

Based on the derived weight $\alpha_{i,t}$, the utility function of node i represents the transmission rate gain above the minimum requirement $R_{i}^{min}$ (the disagreement point). It is defined as

(6) $$U_{i}{(R}_{i,t})=R_{i,t}-R_{i}^{min}$$

By maximizing the Asymmetric Nash Product $\prod {\left(U_{i}\right)}^{\alpha_{i,t}}$, the protocol ensures that nodes with higher $\alpha_{i,t}$ (i.e., higher urgency or congestion) are allocated a larger proportion of the residual channel capacity, effectively mapping the complex sensor states to the optimal rate allocation.

### 3.3. Asymmetric Nash Bargaining Formulation

Traditional Nash Bargaining Solutions assume symmetry, implying all players have equal bargaining power. However, WBAN nodes have heterogeneous QoS requirements. To strictly enforce the priorities defined in Section 3.2.3, we formulate the rate allocation problem using the Asymmetric Nash Bargaining Solution (ANBS). The goal is to maximize the weighted product of the players’ utility gains:

(7) $$\underset{R}{\max}\prod_{i=1}^{N}{\left(R_{i,t}-R_{i}^{min}\right)}^{a_{i,t}}$$

Subject to the following feasibility constraints:

(8) $$R_{i,t}\geq R_{i}^{min}\left(\forall i\in SN\right)$$

(9) $$\sum_{i=1}^{N}R_{i,t}\leq C$$

Here, $R_{i,t}$ is the transmission rate allocated to node i, and C is the total available capacity of the superframe. The exponent $\alpha_{i,t}$ is the dynamic priority weight derived in Equation (5). This formulation ensures that nodes with higher urgency ($\alpha_{i,t}$) require a larger marginal increase in rate to satisfy the maximization condition, effectively prioritizing emergency traffic.

#### 3.3.1. Proof of Convexity

To ensure the proposed solution is globally optimal and computationally tractable, we verify the convexity of the optimization problem. Since the logarithmic function is monotonically increasing, maximizing the product is equivalent to maximizing the sum of logarithms:

(10) $$F\left(R\right)=\ln\left(\prod_{i=1}^{N}{\left(R_{i,t}-R_{i}^{min}\right)}^{\alpha_{i,t}}\right)=\sum_{i=1}^{N}\alpha_{i,t}\ln\left(R_{i,t}-R_{i}^{min}\right)$$

We examine the second-order derivative (Hessian) of the objective function $F\left(R\right)$ with respect to the decision variable $R_{i,t}$:

(11) $$\frac{\partial F}{\partial R_{i,t}}=\frac{\alpha_{i,t}}{R_{i,t}-R_{i}^{min}}$$

(12) $$\frac{\partial^{2}F}{\partial R_{i,t}^{2}}=-\frac{\alpha_{i,t}}{{\left(R_{i,t}-R_{i}^{min}\right)}^{2}}$$

Since priority weights $\alpha_{i,t}>0$ and the square term is always positive, the second derivative is strictly negative ($\frac{\partial^{2}F}{\partial R^{2}}<0$). This confirms that the objective function is strictly concave.

Furthermore, the feasible region is defined by linear inequality constraints ($R_{i,t}\geq R_{i}^{min}$ and $\sum_{i=1}^{N}R_{i,t}\leq C$), which form a convex polyhedron (simplex). Maximizing a strictly concave function over a convex set guarantees the existence of a unique global optimal solution. This mathematically validates the stability of the NBR-MAC protocol.

#### 3.3.2. Verification of Nash Bargaining Axioms

The Nash Bargaining solution requires the utility function to satisfy four axioms: Pareto optimality, Symmetry, Independence of Irrelevant Alternatives (IIA), and Scale invariance.

1. Pareto optimality

Pareto optimality holds if, and only if, no utility improvement for any node is possible without reducing that of at least one other node. In this model, the transmission rate optimization of sensor nodes is constrained by the channel capacity *C*, described by Equation (9). Increasing $R_{i,t}$ for one node necessarily results in a decrease in $R_{j,t}$ for other nodes. Therefore, the utility function satisfies Pareto optimality.

2. Independence of irrelevant alternatives (IIA)

The requirement of independence of irrelevant alternatives states that if certain infeasible rate allocation schemes are removed from the set of options, and the optimal solution remains feasible within the remaining set, then the optimal solution should not change. Let the original optimal solution be

(13) $$R_{i}^{*}=\mathrm{argmax}\prod_{i=1}^{N}\left(U_{i}\left(R_{i,t}\right)-d_{i}\right)$$

Assuming that a non-optimal scheme is removed from the feasible set, since the NBS depends only on the optimal point and constraints, it is not affected by irrelevant schemes. Therefore, the utility function satisfies the independence of irrelevant alternatives.

3. Symmetry

From the utility function defined in Equation (6), it can be seen that if two sensor nodes *i* and *j* have the same parameters, their utility functions satisfy

(14) $$U_{i}\left(R_{i,t}\right)=U_{j}\left(R_{j,t}\right)$$

During the optimization process, since all nodes use the same objective function and constraints, under identical conditions, the two nodes should receive symmetric resource, thereby ensuring that the utility function satisfies the symmetry axiom.

4. Scale invariance

Scale invariance requires that a linear transformation of the utility function does not affect the optimal solution. Let the linear transformation of the utility function be

(15) $$U_{i}^{\prime }\left(R_{i,t}\right)=aU_{i}\left(R_{i,t}\right)+b$$

where a>0. The optimization objective then becomes

(16) $$\underset{V_{i,t}}{\max}\prod_{i=1}^{N}\left({aU}_{i}\left(R_{i,t}\right)+b-d_{i}\right)$$

Since a>0, taking the logarithm yields

(17) $$\underset{V_{i,t}}{max}\sum_{i=1}^{N}\ln\left({aU}_{i}\left(R_{i,t}\right)+b-d_{i}\right)$$

Using the properties of logarithms, we obtain

(18) $$\underset{V_{i,t}}{max}\sum_{i=1}^{N}\left(\ln a+\ln\left(U_{i}\left(R_{i,t}\right)+\frac{b-d_{i}}{a}\right)\right)$$

The constant term lna does not affect the optimization variable. Since a>0 and *b* is a constant, the structure of the optimization objective remains unchanged. Given that the NBS relies solely on the differences in relative utilities, it is evident that this linear transformation does not influence the selection of the optimal solution. The utility function satisfies scale invariance.

#### 3.3.3. Solution of the Bargaining Problem

(19) $$L\left(R,\;\lambda \right)=\sum_{i=1}^{N}\alpha_{i,t}\;\ln\left(R_{i,t}-R_{i}^{min}\right)-\lambda \left(\sum_{i=1}^{N}R_{i,t}-C\right)$$

where λ> 0 is the Lagrange multiplier associated with the capacity constraint. According to the Karush–Kuhn–Tucker (KKT) conditions, the optimal solution must satisfy the stationarity condition, where the partial derivative with respect to each transmission rate $R_{i,t}$ is zero:

(20) $$\frac{\partial L}{\partial R_{i,t}}=\frac{\alpha_{i,t}}{R_{i,t}-R_{i}^{min}}-\lambda =0$$

Rearranging this equation gives the expression for the transmission rate of node i in terms of λ:

(21) $$R_{i,t}=R_{i}^{min}+\frac{\alpha_{i,t}}{\lambda }$$

To determine the value of λ, we sum Equation (21) over all N sensor nodes and apply the channel capacity constraint ($\sum_{i=1}^{N}R_{i,t}=C$):

(22) $$\sum_{i=1}^{N}\left(R_{i,t}-R_{i}^{min}\right)=\sum_{i=1}^{N}\frac{\alpha_{i,t}}{\lambda }=\frac{1}{\lambda }\sum_{i=1}^{N}\alpha_{i,t}$$

and

(23) $$C-\sum_{i=1}^{N}R_{i}^{min}=\frac{1}{\lambda }\sum_{i=1}^{N}\alpha_{i,t}$$

Solving for the Lagrange multiplier λ:

(24) $$\lambda =\frac{\sum_{i=1}^{N}\alpha_{i,t}}{C-\sum_{j=1}^{N}R_{j}^{min}}$$

Finally, by substituting λ back into Equation (21), we obtain the optimal transmission rate allocation for each sensor node i:

(25) $$R_{i,t}^{*}=R_{i}^{min}+\frac{\alpha_{i,t}}{\sum_{k=1}^{N}\alpha_{k,t}}\left(C-\sum_{j=1}^{N}R_{j}^{min}\right)$$

Equation (25) demonstrates that the NBR-MAC protocol allocates resources in two stages:

Every node first receives its minimum required rate $R_{i}^{min}$ ensure basic connectivity and avoid starvation.

Priority-based Surplus Sharing: The residual channel capacity ($C_{res}=C-\sum R_{j}^{min}$ is distributed among nodes proportional to their dynamic priority weights $\alpha_{i,t}$.

This mechanism ensures that nodes with a high data urgency or buffer congestion (large $\alpha_{i,t}$) receive a significantly larger share of the available bandwidth, strictly adhering to the Asymmetric Nash Bargaining axioms.

Complexity Analysis: The proposed NBR-MAC solution (Equation (25)) provides a closed-form expression, which avoids iterative computations typical in gradient-based methods. The computational complexity for calculating the rates of N nodes is $O\left(N\right)$, which is linear and highly efficient for resource-constrained WBAN coordinators. The signaling overhead is minimal, as state variables (buffer, age) are piggybacked on data frames or short beacon requests.

### 3.4. Priority-Based Time Resource Scheduling

This subsection describes the design of the priority-based time-resource scheduling of NBR-MAC protocol, including node priority classification, superframe structure, and the time-slot share. After the optimal transmission rates are determined via the asymmetric Nash Bargaining solution, the superframe structure, node priority classification, and time-slot sharing strategy are designed to translate the optimized rates into concrete MAC-layer scheduling decisions. This hybrid scheduling mechanism integrates guaranteed time-slot allocation with contention-based access, thereby satisfying the stringent latency requirements of high-priority medical data while maintaining fairness among lower-priority traffic.

#### 3.4.1. Node Priority

Sensor nodes in WBANs collect and transmit various types of data during medical monitoring. Sensor nodes can be classified according to the data’s priority. Different types of data have different requirements for delay, reliability, and energy consumption. Considering the importance and real-time requirements of data in actual medical applications, the data transmitted in WBANs are classified into the following three categories:

1. Emergency data (P1)

This includes randomly generated abnormal alarm information or data from medical emergencies, such as arrhythmia alerts and postoperative monitoring data. Once generated, these data often have extremely high requirements for transmission delay and reliability.

2. Periodic data (P2)

This mainly refers to vital sign parameters (such as heart rate, blood pressure, and body temperature) that are periodically collected and reported by sensor nodes. The collection frequency is relatively constant, and they have certain requirements for delay and bandwidth, but not as urgent as emergency data.

3. General data (P3)

This refers to daily health monitoring or auxiliary information with lower sensitivity to delay, such as activity tracking and sleep quality records. These data do not require an immediate response and have a higher tolerance.

#### 3.4.2. Superframe Structure

**Figure 3:**
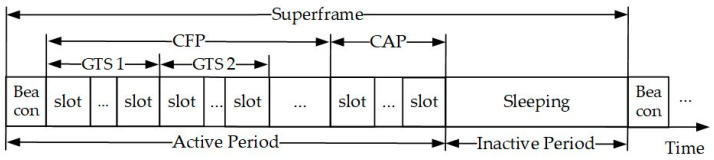
Superframe.

In WBANs, the superframe is typically divided into four phases: beacon, Contention-Free Period (CFP), Contention Access Period (CAP), and inactive period, as shown in Figure 3.

1. Beacon

The coordinator periodically broadcasts a beacon frame to the entire network, synchronizing the clocks of all nodes and providing network management information, such as network status, available channel information, and node join or leave.

2. Contention-Free Period (CFP)

To meet the transmission needs of high-priority or delay-sensitive services, the superframe includes a Contention-Free Period (CFP). Composed of Guaranteed Time Slots (GTS) allocated to specific nodes, the CFP provides exclusive channel access without CSMA/CA contention. This contention-free mechanism ensures high-reliability and low-delay, making it particularly suitable for emergency medical data or data streams with stringent timeliness requirements.

3. Contention Access Period (CAP)

Nodes in this period use a contention mechanism (e.g., CSMA/CA) to compete for channel access rights, suitable for transmitting general or low-priority data. If some nodes are idle or have no data to send, they can choose to sleep during this phase to save energy. Compared with CFP, although contention access may incur collisions, it can fully utilize idle time slots and improve the overall network throughput.

4. Inactive Period

In this phase, nodes can enter a deep sleep state to maximize energy savings, especially suitable for sensor nodes with strict energy constraints. Nodes stop sending or receiving operations during this period and wait for the next superframe to start. The setting of the inactive phase not only extends the network’s lifespan but also reduces unnecessary air interface signal interference.

#### 3.4.3. Time-Slot Sharing

The NBR-MAC protocol dynamically adjusts transmission rates and allocates time slots to sensor nodes to transmit data to the coordinator. According to the status of sensor nodes, including the transmission success ratio, buffer occupancy, buffer age, and data urgency, the transmission rates are calculated using the Nash Bargaining solution in Equation (25).

To translate the optimized transmission rate into practical MAC resources, we define the mapping mechanism between the calculated rate $R_{i,t}^{*}$ and the number of Guaranteed Time Slots (GTS). Let $T_{SF}$ be the superframe duration and $L_{slot}$ denote the effective data carrying capacity of a single time-slot in bits. The number of allocated GTSs $N_{i,t}^{GTS}$ for sensor node i is determined by

(26) $$N_{i,t}^{GTS}=\left\lceil \frac{R_{i,t}^{*}\times T_{SF}}{L_{slot}}\right\rceil$$

where ⌈⋅⌉ denotes the ceiling function. This rounding ensures that the allocated bandwidth meets or slightly exceeds the target rate to satisfy strict reliability requirements. Based on this mapping, nodes with higher urgency (resulting in a higher $R_{i,t}^{*}$) are assigned more time slots in the Contention-Free Period (CFP), effectively prioritizing emergency and periodic traffic over general data.

NBR-MAC employs a priority-aware time-slot sharing strategy that simultaneously ensures timeliness, transmission fairness, and energy efficiency, while providing enhanced transmission guarantees for critical data. The time slots in CFP are allocated to the emergency data (P1) and periodic data (P2) as GTS, in order of priority, with P1 taking precedence. In the case of GTS shortage, the evicted sensor nodes compete in the CAP. For general data (P3) nodes, they only compete for the time slots in the CAP phase through the CSMA/CA mechanism for random access. P3 employs exponential backoff after unsuccessful transmission. Contention window parameters are differentiated by priority level, enabling prioritized channel access. This mechanism ensures that emergency data have higher priority during contention access and experience shorter backoff intervals. Contention window ranges by priority level are listed in Table 2.

The complete resource allocation and scheduling procedure executed at the coordinator is summarized in Algorithm 1.
**Algorithm 1:** NBR-MAC Resource Allocation at CoordinatorInput:    SN = {1, 2,…, N}           // Set of sensor nodes    C            // Total channel capacity per superframe    R_i^min           // Minimum required rate of node i    T_sf            // Superframe duration    S_slot           // Effective payload per time slot Output:    R_i            // Allocated transmission rate for node i    GTS_i            // Number of GTSs allocated to node i 1:  Initialization:2:   For each node i ∈ SN do3:    Initialize buffer length B_i, packet age A_i, urgency level U_i4:    Initialize transmission success ratio TSR_i5:   End for 6:  /* State Collection Phase */7:  At the end of each superframe:8:   Coordinator collects from each node i:9:    - Buffer occupancy B_i10:     - Head-of-line packet age A_i11:     - Urgency indicator U_i12:     - Packet reception ratio TSR_i of previous superframe 13: /* Priority Weight Calculation */14: For each node i ∈ SN do15:    Compute reliability factor:16:     f_i^rel = min(1, TSR_th/TSR_i)17:    Compute buffer congestion factor:18:     f_i^buf = B_i/B_i^max19:    Compute data freshness factor:20:     f_i^age = A_i/A_max21:    Compute medical urgency factor:22:     f_i^urg = U_i23:    Synthesize priority weight:24:     w_i = α·f_i^rel + β·f_i^buf + γ·f_i^age + δ·f_i^urg25: End for 26: /* Asymmetric Nash Bargaining Rate Allocation */27: Compute total residual capacity:28:    C_res = C − ∑_(i∈SN) R_i^min29: Compute sum of priority weights:30:    W = ∑_(i∈SN) w_i31: For each node i ∈ SN do32:    Allocate transmission rate:33:     R_i = R_i^min + (w_i/W) · C_res34: End for 35: /* GTS Mapping and Scheduling */36: For each node i ∈ SN do37:    Map rate to number of GTSs:38:     GTS_i = ceil( (R_i · T_sf)/S_slot )39: End for 40: Sort nodes in descending order of priority (Emergency → Periodic → General) 41: Allocate GTSs in CFP:42:    Assign GTSs to Emergency (P1) and Periodic (P2) nodes first43:    If CFP slots are insufficient:44:     Evicted nodes are scheduled to CAP 45: /* CAP Contention Access */46: Nodes without GTS transmit in CAP using CSMA/CA:47:    Apply priority-dependent contention window parameters 48: /* Inactive Period */49: Nodes without pending data enter sleep mode to save energy 50: End Algorithm

## 4. Performance Evaluation

This section describes the computer simulation and performance analysis of the proposed NBR-MAC protocol for WBANs introduced in the previous section. The Castalia simulation tool within the OMNeT++ platform is used. The proposed algorithm is compared with the IEEE 802.15.6, IEEE 802.15.4, T-MAC, and BOSS. The network performance metrics are the transmission success ratio, packet age, throughput, and energy consumption.

### 4.1. Simulation Scenarios

The simulation employs a star topology, which is typical for WBANs in medical monitoring environments. The network consists of one coordinator and ten sensor nodes. Simulation parameters are listed in Table 3.

To evaluate the adaptability and robustness of the proposed NBR-MAC protocol under different network pressures, three network load levels are set based on two dimensions: data generation rate and the proportion of high-priority traffic. The traffic load types are demonstrated in Table 4.

These three load conditions emulate distinct medical scenarios, where an increasing node load amplifies both data volume and slot competition, comprehensively evaluating the protocol.

To explicitly simulate the dynamic environment of WBANs, we incorporated a Log-distance path loss model combined with lognormal shadowing (standard deviation σ=4.0 dB) to reflect body-shadowing effects. The channel quality $\rho_{i,t}$ in the utility function is updated periodically based on the Packet Reception Ratio (PRR) of the previous superframe.

Furthermore, to ensure statistical validity, all simulation results are averaged over 50 independent runs with different random seeds. The results presented in the following sections represent the mean values, and the variance was observed to be within a 5% margin, ensuring the reliability of the performance comparison.

All simulation results represent the average of 50 independent runs to ensure statistical validity, with 95% confidence intervals calculated to verify that the margin of error remains within ±2% of the mean values.

### 4.2. Simulation Results and Analysis

#### 4.2.1. Transmission Success Ratio

Transmission Success Ratio (TSR) is a reliability metric for the communication link, defined as the ratio of successfully transmitted data to the total amount of data attempted for transmission. It is influenced by channel state, interference level, network load, and transmission mechanisms.

**Figure 4:**
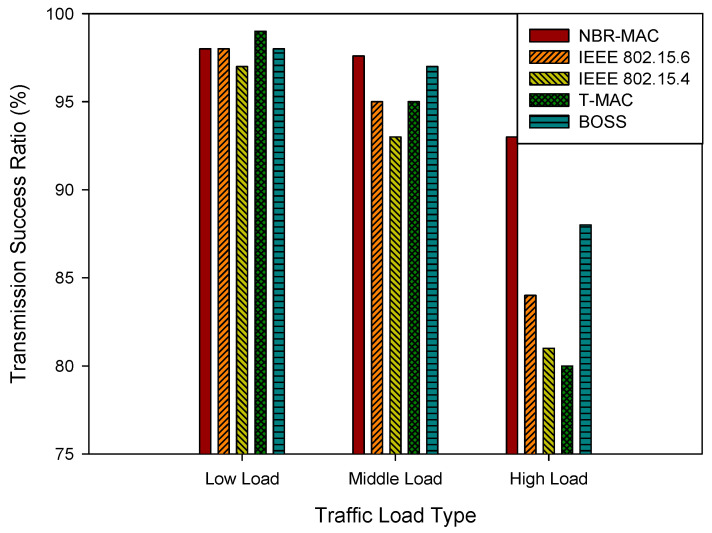
Transmission success ratio under three traffic load types.

Figure 4 depicts the TSR comparation of the proposed MAC with other four schemes under three traffic load types. Notice that the TSR of all protocols is greater than 95% under low-load conditions, indicating that when network resources are relatively abundant, all protocols can provide moderately reliable data transmission services. However, as network load increases, the performance differences among the protocols gradually become apparent. The NBR-MAC protocol achieves approximately 97.6% and 93% transmission success ratio under middle and high loads, respectively, significantly outperforming protocols such as IEEE 802.15.4, T-MAC, and BOSS. This is due to the dynamic scheduling strategy of the NBR-MAC protocol, which can respond in real-time to changes in network conditions and prioritize the transmission needs of emergency and periodic data, thereby effectively reducing the probability of data collisions and packet loss. In contrast, IEEE 802.15.4 and T-MAC protocols lack dynamic adjustment mechanisms and cannot timely alleviate network congestion under high-load conditions, leading to a significant decrease in the transmission success ratio.

#### 4.2.2. Packet Age

We define the Packet Age (PA) as the time interval from data packet generation to successful reception. The calculation formula is as follows:

(27) $${PA}_{i}\left(t\right)=t-\mu_{i}$$

where *t* is the arrival time of data packet *i*, and $\mu_{i}$ is the generation timestamp of packet *i*. The average packet age represents overall data timeliness.

**Figure 5:**
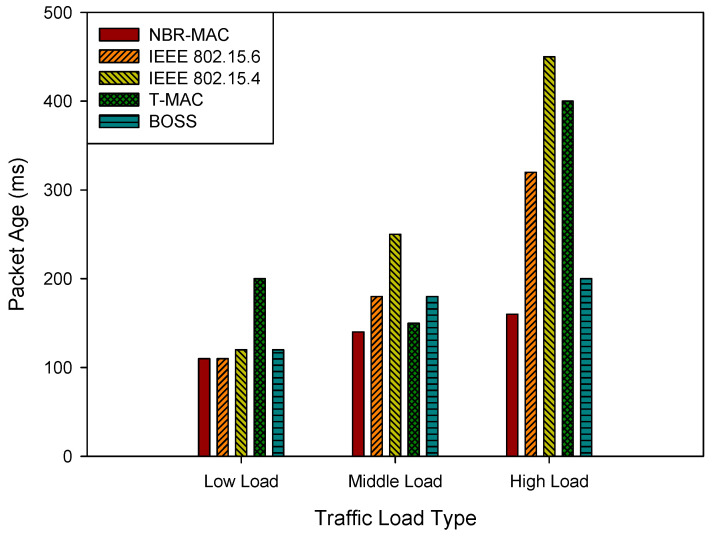
Average packet age under three traffic load types.

Figure 5 demonstrates the simulation results of the average packet age under three traffic load types. As shown in the figure, with the increase in network load, the PA of all protocols shows varying degrees of upward trends. Among them, the PA of IEEE 802.15.4 increases sharply under middle- and high loads, reaching significantly high values, indicating that its data transmission is severely delayed and cannot meet the needs of real-time monitoring. In contrast, NBR-MAC maintains a relatively low PA under all load conditions. This is mainly due to its dynamic adjustment of data transmission rates and exclusive time-slot allocation mechanism for high-priority data, which effectively reduces data waiting time and enhances the timeliness and reliability of critical data.

#### 4.2.3. Throughput

Throughput refers to the number of bits successfully received per unit time. The calculation formula is as follows:

(28) $$Throughput=\frac{total\;successfully\;delivered\;bits\;in\;\Delta t}{\Delta t}$$

**Figure 6:**
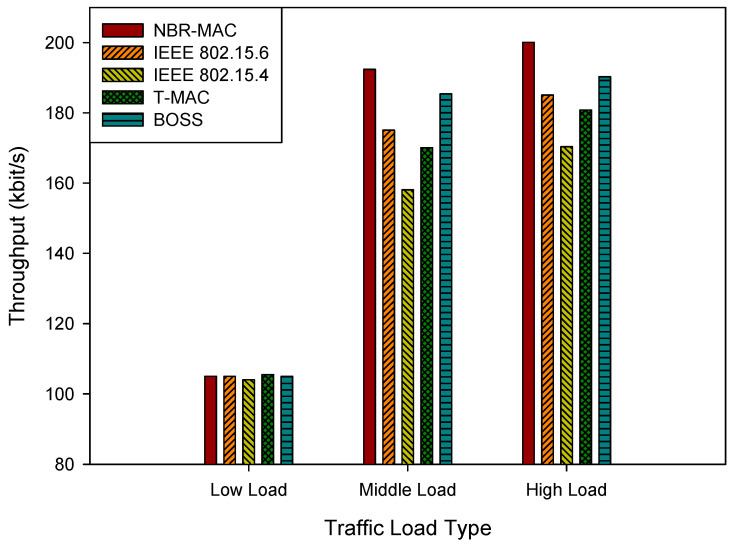
Throughput under three traffic load types.

Figure 6 shows the simulation results of throughput under three traffic load types. At the low load type, throughput is comparable across protocols. However, as traffic increases, performance gaps widen and NBR-MAC establishes a clear advantage, particularly under high-load conditions. The throughput of NBR-MAC and BOSS is relatively close. This is because BOSS employs a slot allocation mechanism based on a cooperative game, enabling nodes to utilize channel resources more efficiently. However, BOSS lacks a contention phase, resulting in poorer flexibility and fairness. In contrast, NBR-MAC, through its dynamic rate adjustment mechanism, can more flexibly adapt to different load conditions. Moreover, by combining guaranteed time slots (GTS) with contention access (CAP), NBR-MAC not only ensures the needs of high-priority nodes but also takes into account the requirements of low-priority nodes.

#### 4.2.4. Energy Consumption

**Figure 7:**
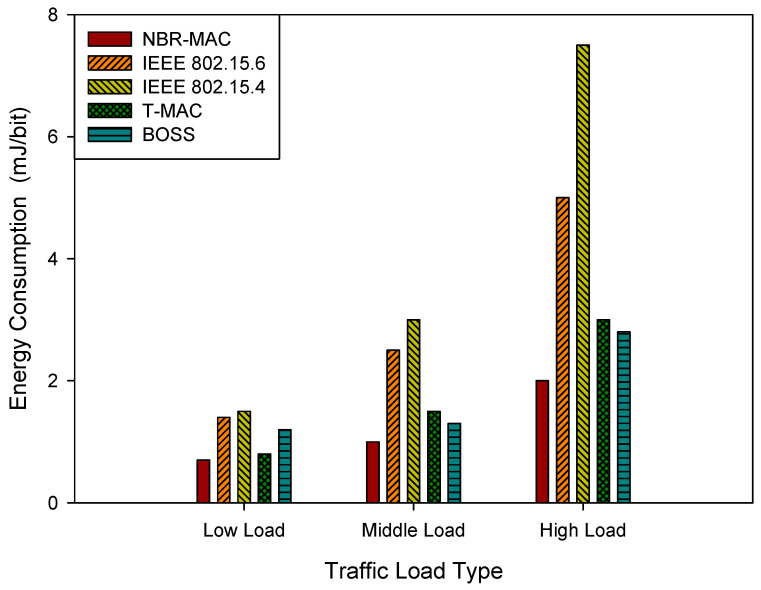
Energy consumption under three traffic load types.

We measure the energy consumption and then compare the average energy consumed for successfully transmitting per bit, which is calculated as follows:

(29) $$E_{avg}=\frac{\sum_{i=1}^{N}E_{i}}{\sum_{i=1}^{N}D_{i}}$$

where $E_{i}$ is the total energy consumed by node *i*, and $D_{i}$ is the amount of data successfully transmitted by node *i*. In WBANs, energy consumption directly affects the lifecycle of nodes, especially for energy-constrained medical sensor devices. The simulation results of energy consumption per bit under three traffic load types are demonstrated in Figure 7.

Under the low load type, energy consumption remains low across protocols. However, it rises markedly with load due to increased transmission and contention costs. NBR-MAC achieves the lowest energy consumption across all loads, attributed to its Nash Bargaining model that jointly optimizes transmission rates, GTS allocation, and CAP contention. IEEE 802.15.4 exhibits a steep energy-consumption increase under middle and high loads due to its limited energy control, which accelerates node depletion and shortens network lifespan. T-MAC and BOSS exhibit intermediate energy consumption, higher than NBR-MAC but lower than IEEE 802.15.4.

The proposed protocol increases the computational complexity and signaling overhead.

## 5. Conclusions

This paper proposes NBR-MAC, a hybrid MAC protocol for WBAN transmission optimization in complex environments based on a cooperative game. The transmission rate is adjusted to match network load dynamics, while node prioritization and strategic time-slot sharing reduce collisions, guarantee emergency data timeliness, and enhance stability. OMNeT++ simulations validate that NBR-MAC improves network performance, including transmission success ratio, packet age, throughput and energy consumption across load conditions, and significantly enhances WBAN reliability and timeliness. Future work will address NBR-MAC’s computational overhead from Nash Bargaining and validate its scalability on real WBAN testbeds under realistic mobility patterns.

## Figures and Tables

**Table 1:** Comparison of MAC Protocols in WBAN.

Protocol	Access Mechanism	Optimization Goal	Priority Support	Mathematical Model
IEEE 802.15.6	Hybrid (CSMA + Polling)	Reliability/Power	Static (UP levels)	None
T-MAC	Contention-based	Energy/Duty-cycle	Low	Adaptive Duty Cycle
BOSS	TDMA	Slot Allocation	Middle	Standard Nash Bargaining
NBR-MAC	Hybrid (GTS + CSMA)	Rate & Latency	Dynamic (Urgency)	Asymmetric Nash Bargaining

**Table 2:** Contention window ranges by priority level.

Priority	Data Category	CWmin	CWmax
P1	Emergency Data	1	4
P2	Periodic Data	2	8
P3	General Data	4	8

**Table 3:** Simulation parameters.

Parameter	Value
Simulation Time	100 s
Simulation Area	10 m × 10 m
Number of Nodes	11
Channel Fading Model	Log-distance Path Loss Model
Data Generation Rate	3–12 pkt/s
Packet Size	128–512 B
Frequency Band	2400–2483.5 MHz
Superframe Order	5
Slot Duration	1.024 ms
Queue Size	32 packets
Transmission Energy	36.5 mW
Reception Energy	41.4 mW
Sleep Energy	42 μW
High-Priority Traffic Ratio	10–30%

**Table 4:** Traffic load types.

Priority	Data Generation Rate	Proportion of High-Priority Traffic
Low Load	3–5 pkt/s	10%
Middle Load	5–8 pkt/s	20%
High Load	8–12 pkt/s	30%

## Data Availability

Data is contained within the article.

## Abbreviations

The following abbreviations are used in this manuscript:

WBAN	Wireless Body Area Network
LR-WPAN	Low-Rate Wireless Personal Area Network
IoT	Internet of Things
MAC	Medium Access Control
QoS	Quality of Service
UAV	Unmanned Aerial Vehicle
NBR-MAC	Nash Bargaining Rate-optimization MAC
CFP	Contention-Free Period
CAP	Contention Access Period
GTS	Guaranteed Time Slot
